# Indoor collections of the *Anopheles funestus *group (Diptera: Culicidae) in sprayed houses in northern KwaZulu-Natal, South Africa

**DOI:** 10.1186/1475-2875-6-30

**Published:** 2007-03-14

**Authors:** Joel C Mouatcho, Keith Hargreaves, Lizette L Koekemoer, Basil D Brooke, Shüne V Oliver, Richard H Hunt, Maureen Coetzee

**Affiliations:** 1 Vector Control Reference Unit, National Institute for Communicable Diseases, NHLS, Private Bag X4, Sandringham, Johannesburg 2131, South Africa; 2 Division of Virology and Communicable Diseases Surveillance, School of Pathology of the National Health Laboratory Service and the University of the Witwatersrand, Johannesburg, South Africa; 3 Malaria Control Programme, KwaZulu-Natal Department of Health, Jozini, South Africa; 4 School of Animal, Plant and Environmental Sciences, University of the Witwatersrand, Johannesburg, South Africa

## Abstract

**Background:**

Insecticide resistance in malaria vector mosquitoes presents a serious problem for those involved in control of this disease. South Africa experienced a severe malaria epidemic during 1999/2000 due to pyrethroid resistance in the major vector *Anopheles funestus*. Subsequent monitoring and surveillance of mosquito populations were conducted as part of the malaria vector control programme.

**Methods:**

A sample of 269 *Anopheles funestus s.l. *was collected in Mamfene, northern KwaZulu-Natal, using exit window traps in pyrethroid sprayed houses between May and June 2005. Mosquitoes were identified to species level, assayed for insecticide susceptibility, analysed for *Plasmodium falciparum *infectivity and blood meal source.

**Results:**

Of the 220 mosquitoes identified using the rDNA PCR method, two (0.9%) were *An. funestus s.s. *and 218 (99.1%) *Anopheles parensis*. Standard WHO insecticide susceptibility tests were performed on F1 progeny from wild caught *An. parensis *females and a significant survival 24 h post exposure was detected in 40% of families exposed to 0.05% deltamethrin. Biochemical analysis of F1 *An. parensis *showed no elevation in levels/activity of the detoxifying enzyme systems when compared with an insecticide susceptible *An. funestus *laboratory strain. Among the 149 female *An. parensis *tested for *P. falciparum *circumsporozoite infections, 13.4% were positive. All ELISA positive specimens (n = 20) were re-examined for *P. falciparum *infections using a PCR assay and none were found to be positive. Direct ELISA analysis of 169 blood meal positive specimens showed > 75% of blood meals were taken from animals. All blood fed, false positive mosquito samples for the detection of sporozoites of *P. falciparum *were zoophilic.

**Conclusion:**

The combination of pyrethroid resistance and *P. falciparum *false-positivity in *An. parensis *poses a problem for vector control. If accurate species identification had not been carried out, scarce resources would have been wasted in the unnecessary changing of control strategies to combat a non-vector species.

## Background

Historical studies have shown that *Anopheles funestus *and *Anopheles arabiensis *are the principal malaria vectors in southern Africa [1,2]. The *Anopheles funestus *group includes nine African species: *An. funestus*, *Anopheles rivulorum*, *Anopheles vaneedeni*, *Anopheles leesoni*, *Anopheles confusus, Anopheles fuscivenosus, Anopheles brucei*, *Anopheles parensis *and *Anopheles aruni*. Recently, "*An. rivulorum*-like" was added to this group based on molecular sequencing data [3]. These species show morphological overlap, although some species can be identified on egg and larval characteristics [1,2]. Of these, *An. funestus *is the only member of the group that is recognized as an important vector of malaria in Africa, with *An. rivulorum *only a minor vector at a localized site in Tanzania [4]. *Anopheles vaneedeni *was experimentally infected in the laboratory with *Plasmodium falciparum *but has not been implicated in malaria transmission in nature [5].

**Figure 1:**
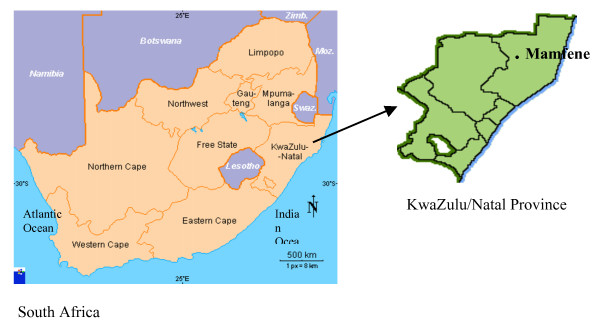
Map of South Africa showing the collection site, Mamfene, within KwaZulu-Natal.

**Figure 2:**
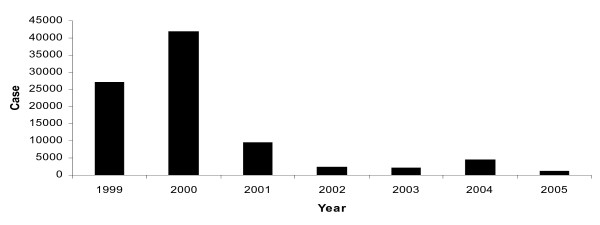
Reported malaria cases in KZN (1999–2005). Data were obtained from the Jozini Department of Health (unpublished).

Mamfene village (Figure 1), in northern KwaZulu-Natal Province, South Africa, is situated in an area that was worst affected by the malaria epidemic of 1999/2000 [6]. The seven-fold increase of malaria cases compared with 1994 was mainly linked to the recurrence of *An. funestus *which was shown to have developed resistance to pyrethroid insecticides [6,7]. A policy decision to reintroduce DDT for malaria control in traditional style houses whilst continuing to use pyrethroids in western style houses resulted in a significant decrease in the number of reported malaria cases in the region during the period 2001 to date (Figure 2). Continued monitoring of vectors within this region has subsequently demonstrated DDT resistance in *An. arabiensis *(another major malaria vector) and *Anopheles quadriannulatus *(a non vector) during 2002 [8]. The latter two species are both members of the *Anopheles gambiae *complex.

Monitoring of malaria vector species continued during 2005 as support for the malaria control programme in northern KwaZulu-Natal. Field collections produced large numbers of the *An. funestus *group collected in pyrethroid sprayed houses in Mamfene.

The aim of this study, therefore, was to determine the species composition of the *An. funestus *group in Mamfene, the *P. falciparum *infection rates in these species, their feeding behavior and their susceptibility to insecticides.

## Materials and methods

### Study site

Mosquitoes were collected in Mamfene, northern KwaZulu-Natal, South Africa (27°23' S, 32°12' E). Mamfene is an undeveloped rural and rice growing area near the Balamhlanga river that forms an extensive marsh during the rainy season (October to February). Marshy conditions may persist during the dry season when the rice paddies upstream are drained. In addition to growing rice, inhabitants of this area also grow vegetables and cotton, and keep livestock for subsistence. Two types of houses are found in the area: traditional houses, which are mud structures, and western-style houses built using cement and bricks. During 2005, vector control was conducted by spraying a combination of DDT in traditional houses and pyrethroid (deltamethrin) in the westernized houses.

### Mosquito collections

Window exit traps were used to collect adult mosquitoes indoors between May and June 2005. Collections were conducted by the KwaZulu-Natal Department of Health, Malaria Control Programme teams. Live mosquitoes were identified using morphological keys [1]. Mosquitoes identified as *An. funestus *group were transported to the National Institute for Communicable Diseases (NICD) in Johannesburg. Live, blood-fed female anophelines were individually isolated for oviposition and kept under standard insectary conditions (25°C, 75–80% relative humidity and 12 hrs light: dark with 45 min dusk/dawn transition). Larvae were reared through to adults. The F1 progeny (1–4 days old) were subjected to standard WHO insecticide susceptibility tests [9]. Unexposed males and females (1–4 day old) from large families were frozen at -70°C for biochemical analysis. Dead wild-caught females were preserved on silica gel for species identification and sporozoite detection by ELISA.

### Species-specific identification

Wild-caught mosquitoes were individually identified using PCR [10]. Two legs per mosquito were used for the assay.

### Sporozoite detection by indirect ELISA

The heads and thoraces of wild female mosquitoes were tested for the presence of *P. falciparum *circumsporozoite protein (CSP) using monoclonal antibodies 2A10 [11]. Seven negative controls and one positive control were included on each plate. The negative controls consisted of triturated, unfed *An. funestus s.s. *colonized in the Botha De Meillon Insectary at the NICD. The positive control consisted of a synthetic peptide standardized against *P. falciparum*. Results were analysed using a microtitre plate reader at a wavelength of 405 nm. The absorbance cut off value for positive specimens was calculated as twice the mean value of the four negative controls. Specimens that tested positive for *P. falciparum *were re-examined using PCR [12] in order to confirm the results.

### Polymerase chain reaction (PCR) in order to confirm indirect ELISA positive specimens

Phenol-chlorophorm was used to extract DNA from the positive ELISA homogenate [13]. Two *Plasmodium- *specific primers based on the sequence of the small subunit ribosomal RNA (ssrRNA) namely rPLU (5 and 6) and rFAL (1 and 2) were used [12]. The rPLU and rFAL primers were separately used for the first and second round of PCR. Briefly, 1 μl was used for PCR [12] with the following conditions: 95°C for 5 min followed by 25 cycles at 94%°C for 1 min, 58°C for 2 min, 72°C for 2 min and a final auto-extension at 72°C for 5 min. One microlitre of the first round PCR was used as template for the second PCR.

### Mosquito blood sources (direct ELISA)

Half-gravid and fully fed mosquitoes that were not used for egg-laying were preserved on silica gel. These were screened for blood meal source using direct ELISA method [14]. All specimens were screened for human, bovine, chicken, goat and sheep blood. Absorbance at 405 nm was recorded with an ELISA plate reader 30 min after the addition of the substrate. Samples were considered positive if absorbance values exceeded the mean plus three times the standard deviation of four negative controls (unfed colony mosquitoes).

### Insecticide susceptibility tests

Standard insecticide treated papers supplied by WHO [9] were used to test for susceptibility/resistance to 0.1% bendiocarb, 4% DDT, 0.05% deltamethrin and 0.75% permethrin. *Anopheles quadriannulatus *(SKUQUA), known to be susceptible to all insecticides, was used as a control to ensure the reliability of each batch of impregnated papers. Negative controls consisted of untreated papers. Knockdown was recorded after 1 h and a 10% sucrose solution was made available to survivors. Final mortality was scored 24 h post exposure.

### Biochemical analysis

Assays designed to quantify average levels of monooxygenase, non-specific esterase and glutathione-S- transferase (GST) activity as well as to detect the presence, in individual mosquitoes, of an altered acetylcholinestrase associated with carbamate/organophosphate resistance were performed [15]. Propoxur was used as a reference carbamate insecticide for the acetylcholinesterase assay. Mixed samples of male and female F1 progeny from familial material initially stored at -70°C were assayed concurrently with male and female samples drawn from an *An. funestus *laboratory strain (designated FANG) which is fully susceptible to insecticides. Optical densities were directly compared between the *An. parensis *and FANG samples by gender following adjustment for total protein content.

## Results

### Mosquito collections

**Figure 3:**
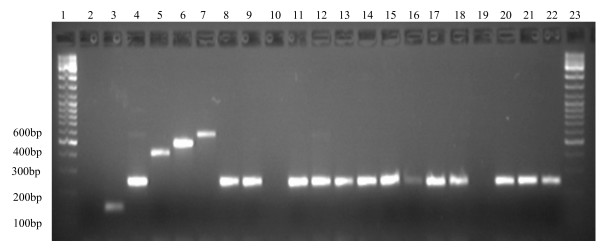
**Species identification of members of *An. funestus *group from Mamfene. **Lanes 1&23: 100 bp (HyperLadder IV from Bioline); Lane2: negative control; Lanes 3–7: positive controls with 3: *An leesoni *146 bp, 4: *An. parensis *252 bp, 5: *An. rivulorum *411 bp, 6: *An. funestus *505 bp and 7: *An. vaneedeni *587 bp. Lanes 8–9, 11–18 and 20–22 *An. parensis *from Mamfene. Lanes 10 and 19 did not amplify.

A total of 269 mosquitoes morphologically identified as members of the *An. funestus *group were collected from window exit traps. Of these, 220 were identified to species level by PCR. Two species were identified: *An. parensis *99.1% (n = 218) and *An. funestus s.s *0.9% (n = 2). The remaining specimens (n = 49) could not be identified despite three attempts, although the positive controls amplified successfully (Figure 3).

### Sporozoite detection

Of 149 female *An. parensis *assayed using the ELISA method, 13.4 % (20/149) tested positive for *P. falciparum *circomsporozoites. Those females identified as *An. funestus *(n = 2) were negative. This assay was repeated three times and the results concurred. Owing to this abnormally high infection rate, all positive specimens were retested using a PCR-based method [12] in an effort to confirm these results. None of the specimens re-examined showed *P. falciparum *amplicons even though the positive control amplified successfully with each reaction.

### Blood meal identification

**Figure 4:**
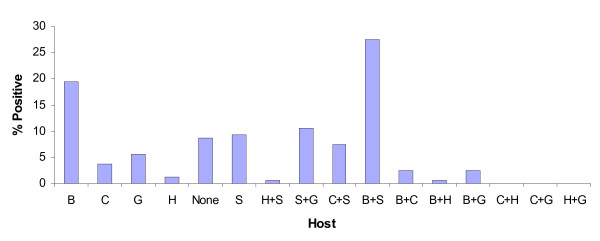
**Percentages of different and mixed blood meals for *Anopheles parensis *from Mamfene**. B = bovine, C = chicken, H = human, G = goat and S = sheep.

A total of 169 mosquitoes identified by PCR (167 *An. parensis *& 2 *An. funestus*) had taken blood meals prior to collection. Both the *An. funestus *specimens had taken bovine blood. Results for the *An. parensis *sample are summarized in Figure 4 which shows a broad spectrum of blood meal sources. These include: bovine 19.37%, sheep 9.37% goat 5.62%, chicken 3.75% and human 1.25%. Multiple blood meal sources were detected in *An. parensis*: 27.5% were positive for bovine and sheep blood and only 0.62% was positive for human and goat blood. Identification of each source of blood was done in duplicate. The remaining 8.75% of blood fed *An. parensis *could not be assigned a blood meal source.

Of the 13 % of females that tested positive for the presence of *P. falciparum *sporozoites, 65 % (13/20) were blood fed. Identification of blood meal source of those females by direct ELISA showed that 53.85 % were positive for bovine blood, 38.46 % for sheep blood and 7.69 % had a mixed blood meal source.

### WHO susceptibility tests

**Figure 5:**
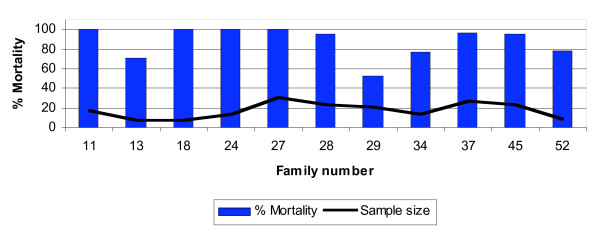
Percentage mortality 24 h post exposure to0.05% deltamethrin of F1 progeny from *An. parensis *families.

Table 1 shows the percentage mortality recorded 24 h post exposure of 1–4 day old F1 progeny of wild caught *An. parensis *females. They were assayed against four insecticides: two pyrethroids (deltamethrin and permethrin), one carbamate (bendiocarb) and one organochlorine (DDT). These samples were susceptible to permethrin, bendiocarb and DDT but showed signs of resistance to deltamethrin. Significant survival 24-hr post exposure (> 20%), was detected in 4 of 11 families exposed to 0.05% deltamethrin (Fig. 5) with one family (#29) giving 47.6% survival. All positive controls were 100% susceptible to the insecticides and all negative controls on untreated papers survived.

**Table 1:** Results of insecticide susceptibility tests on F_1 _progeny from families of wild-caught *Anopheles parensis *24 hrs post-exposure to three classes of insecticides.

**Insecticide**	**No. families**	**TotalNo.**	**Alive**	**Dead**	**% Mortality**
0.1% Bendiocarb	10	205	4	201	98.05
4% DDT	4	81	0	81	100
0.75% Permethrin	6	118	2	116	98.31
0.05% Deltamethrin	11	204	30	174	85.29
Control		100	0	100	100

Control = 25 susceptible laboratory *Anopheles quadriannulatus *colony mosquitoes exposed to each insecticide.

### Biochemical analysis

Direct comparison of optical density values between the *An. parensis *and laboratory *An. funestus *male and female samples are summarized in Table 2. Two-sample t tests assuming equal variances revealed no significant differences in detoxifying enzyme levels between strains by gender with the exception of the α esterase assay in which the *An. funestus *female sample showed a significantly higher mean optical density (OD) value compared to the corresponding *An. parensis *sample. Male (n = 24) and female (n = 24) samples from both strains showed > 55% inhibition of acetylcholinesterase activity when challenged with propoxur with the exception of one *An. parensis *female that showed 33.3% inhibition.

**Table 2:** Mean optical density values for glutathione S-transferase (GST), monooxygenase (Oxy) and esterase (Est) enzymes using α and β napthyl acetate as substrates.

		n	GST	Oxy	αEst	βEst
Females	*An. parensis*	24	0.024 (0.003)	0.335 (0.022)	0.134 (0.018)	0.115 (0.016)
	*An. funestus *(FANG)	24	0.022 (0.002)	0.324 (0.016)	0.201 (0.026)	0.16 (0.021)
	p		0.73	0.69	0.04	0.1
Males	*An. parensis*	24	0.024 (0.003)	0.245 (0.007)	0.173 (0.023)	0.178 (0.017)
	*An. funestus *(FANG)	24	0.024 (0.002)	0.246 (0.01)	0.207 (0.027)	0.14 (0.018)
	p		0.86	0.94	0.35	0.16

"n" = sample size; Figures in parentheses are the standard errors for each sample; p indicates significance in difference between *An. parensis *and *An. funestus *samples by enzyme system by gender following 2-sample t tests.

## Discussion

Little is known of the vectorial capacity of *An. parensis *and it is generally regarded as zoophilic with no medical importance [1,2]. *Anopheles parensis *is found in eastern Africa from Kenya and Tanzania in the north to KwaZulu-Natal Province in South Africa [1,2]. In KwaZulu-Natal, *An. parensis *has previously been found resting indoors in formerly insecticide sprayed localities (insecticide not mentioned, but DDT traditionally used for house spraying in South Africa) [2]. More recent work in the same area [7,10] also found *An. parensis *resting inside insecticide sprayed houses. In both these studies failure to identify a proportion of *An. funestus *group collections could be the result of DNA degradation due to poor preservation or the current lack of species specific primers for all members of this group. The PCR assay [10] only identifies the five most common members and cannot exclude the possibility that other members of the group might be amongst the specimens that failed to be identified. It is suggested that unidentified *An. funestus *group specimens from Kenya (10%) could have been *An. confusus *[16]. *Anopheles confusus *adults are morphologically similar to *An. funestus s.s. *[2] but cannot be identified to species level using current molecular methods. This rare species has been recorded in Zimbabwe but is generally confined to the plateau areas of eastern Africa from Kenya to South Africa [2].

Recent studies in the Mwea area of central Kenya showed high numbers of *An. parensis *resting inside houses [16]. Of these only 1.4% had fed on human blood and none were sporozoite positive [16]. The present study showed that 13.5% of the specimens tested were positive for *P. falciparum *circumsporozoite protein using monoclonal antibodies [11] but only 1.25% had fed on humans. Re-analysis using PCR [12] revealed no positive specimens in this sample. False positive results have been recorded previously [17-19]. A cross-reactive factor (s) in plasma from swine and bovine blood has been postulated to interfere with the ELISA technique resulting in false positives in *An. dirus *when testing for *P. falciparum *and *P. vivax *sporozoites [19]. False positive results have also been reported in *An. gambiae *tested for *P. falciparum *antigens [18] and in *An. marshallii s.l. *[17].

None of the 65 % (13/20) false positive *An. parensis *specimens that were blood fed had fed on humans. The remaining 35% of positives (7/20) did not show any indication of a blood meal, but still gave false positive results. These false positives could be due to traces of animal blood accumulating in the haemolymph [19]. Why the South African *An. parensis *should give high false positive results but the Kenyan samples did not [9] needs further investigation.

F1 Progeny from the *An. parensis *families were susceptible to DDT, permethrin and bendiocarb but showed significant resistance to 0.05% deltamethrin according to WHO criteria [9] in some families, with an overall 24-hr mortality of 85.3%. However, preliminary biochemical analysis showed no detoxifying enzyme systems that could be associated with deltamethrin resistance.

## Conclusion

The combination of insecticide resistance and false positive *P. falciparum *infections in *An. parensis *underlines yet again the importance of accurate species identification in a vector control monitoring and surveillance programme. Without accurate species identification, scarce resources would have been wasted on changing vector control intervention strategies to target a species that is not feeding on humans and is not transmitting malaria parasites. Eighty years after Watson coined the term "species sanitation" to describe the process of targeting only disease transmitting vectors [20], the concept remains as valid today as it did in those early days of malaria control.

## Authors' contributions

JC carried out the rearing of mosquitoes, all the bioassays including ELISAs and molecular studies, wrote the first and subsequent drafts of the manuscript.

K took charge of the field work and morphological identification of anophelines.

RH assisted in interpretation of bioassay results and field work data.

SV performed the biochemistry assays.

BD interpreted the results of the biochemistry assays and contributed to the subsequent writing of the manuscript.

LL and M conceived the project, oversaw its implementation and contributed to the subsequent writing of the manuscript.

All authors read and approved the final manuscript.
